# Supplementary material for: Are carbon emissions trading and green financial instruments synergistic? -Comprehensive quantitative research based on content analysis
Source: PLoS One. 2024 Mar 7;19(3):e0298601. doi: 10.1371/journal.pone.0298601 (PMC10919601; doi:10.1371/journal.pone.0298601)
Supplement: S1 Data — (ZIP) [file pone.0298601.s001.zip › Data/地级市Z_F工作报告.zip!/╣π╢1⁄2/╗▌╓▌╩╨/└┤╫╘╣1⁄2╓┌║┼í╛┬φ┐╦╩2╛▌═°í┐_2011.docx]

各位代表：

　　我代表市人民政府向大会作报告，请连同《惠州市国民经济和社会发展第十二个五年规划纲要（草案）》一并审议，并请各位政协委员和列席人员提出意见。

　“十一五”时期及2010年工作回顾

　　“十一五”时期是我市发展史上极不平凡的五年，是我市经济社会发展最快、城乡面貌变化最大、人民得到实惠最多的五年。五年来，在省委、省政府和市委的正确领导下，在市人大及其常委会和市政协的监督支持下，市政府坚持以邓小平理论、“三个代表”重要思想为指导，深入贯彻落实科学发展观，全面贯彻党的十七大及各次全会精神，认真落实党中央、国务院和省委、省政府各项决策部署，积极实施《珠江三角洲地区改革发展规划纲要》（以下简称“《规划纲要》”），顽强战胜前进道路上的各种困难，全力克服国际金融危机的不利影响，大力推进“三促进一保持”，坚定不移调结构，脚踏实地促转变，圆满完成市十届人大五次会议确定的各项任务和“十一五”规划的各项目标。全市经济社会发展实现了八个“新跨越”：

　　一、经济持续强劲发展，综合实力提升实现新跨越

　　经济总量五年翻一番。2010年，全市生产总值1730亿元，比上年（下同）增长18%，比2005年翻一番，五年（下同）年均增长15.4%。人均生产总值4.34万元，年均增长13.5%，进入中等收入地区和经济增速最快城市行列。

　　财税金融大幅增长。全市财税总收入597.9亿元，增长32.9%，年均增长27.7%，地方财政一般预算收入131.2亿元，增长29.2%，年均增长30.5%，分别是2005年的3.4倍和3.8倍，财政实力与经济总量同居全省第6位。全市金融机构本外币存款余额2087.5亿元，贷款余额1224亿元，分别是2005年末的2.5倍和3倍，存贷比达到58.6%。

　　发展动力显著增强。全社会固定资产投资894亿元，增长17.8%，五年累计完成投资3037.4亿元，是“十五”时期的2.8倍；社会消费品零售总额582.5亿元，增长19%，年均增长18.3%；外贸出口202.3亿美元，增长18%，年均增长13.7%。“惠货全国行”取得明显成效，签订销售合同金额超千亿元。产品内外销比例调整为59.7︰40.3，形成了内外需协调拉动经济增长的新格局。

　　二、结构调整扎实推进，产业优化升级实现新跨越

　　三次产业结构由2005年的9.3︰56.7︰34调整为6︰59︰35，产业结构高级化、适度重型化进程加快，产业集聚效应日益突显，五大基地建设呈现迅猛发展的强劲态势。

　　炼化一体石化产业链形成。中海壳牌南海石化、中海油1200万吨炼油两大龙头项目建成投产，并带动60个石化中下游项目落户大亚湾，总投资超过1400亿元，形成了规模化发展的石化产业集群，大亚湾石化区成为中国石化工业示范园区，精细化工园区建设快速推进。石化产业实现增加值303.1亿元，增长41.2%，实现年均62.5%的高速增长，石化产业成长为我市新兴支柱产业。

　　电子信息产业向高端发展。TCL液晶模组整机一体化、科锐LED芯片等项目建成投产，新型显示、新一代通信等16个项目入选省高端新型电子信息产业重大项目，TCL、德赛、华阳、侨兴、光弘5家企业入选中国电子信息百强企业。电子信息产业实现增加值281.5亿元，增长18.7%。

　　新兴产业发展势头迅猛。光电产业基地列入省第一批战略性新兴产业基地。全市29家企业37个项目列入省现代产业500强，总投资1000亿元，其中已有32个项目开工建设，完成投资160亿元。清洁能源产业规模快速扩张，LNG电厂一期、平海电厂一期、国华热电联供、抽水蓄能电站5台机组建成投产，新增装机容量532万千瓦。

　　现代服务业向优质发展。旅游业发展“3510”工程深入实施，西湖、罗浮山等旅游景区建设和环大亚湾五星级酒店群建设全面推进，五年新增五星级酒店4家、4A级旅游景区7个。龙门县成为全国首批“中国温泉之乡”。全年接待游客2501万人次、旅游总收入140.8亿元，分别增长15.2%、22.3%。金融业蓬勃发展，五年引进银行10家、证券保险机构42家，扶持企业上市4家，荣获“中国金融生态市”称号。房地产业平稳健康发展，商贸、物流、会展、传媒等现代服务业加速发展。

　　园区经济强力推进。大亚湾石化区规划面积由27.8平方公里扩展到65平方公里，全区经济年均增长38.3%，石化产业的龙头地位进一步强化。仲恺高新区完成体制机制改革创新，管理范围由12.5平方公里扩展到320平方公里，成为国家电子信息产业基地、通讯产业基地、激光头产业基地和视听产品产业园，集聚高新技术产业的能力进一步增强。东江高新科技产业园建设高标准、高起点、高效率推进，引进项目20多个，总投资超过120亿元，成为全市经济的又一重要增长极。

　　科技创新能力持续增强。五年累计新增省级以上工程技术研发中心24家、重点实验室3家，建立产学研结合示范基地5个，国家石油石化产品质量监督检验中心落户我市。获专利授权4991项、中国专利金奖1项、省科技进步奖24项，连续三届蝉联全国科技进步先进市，列入国家知识产权试点城市。科技进步对工业经济增长贡献率达56%，全市高新技术产品产值1600亿元，是“十五”期末的2.4倍。

　　三、基础设施升级换代，现代城市发展实现新跨越

　　以建设广东最优美生态宜居城市为目标，大力实施“南进北拓、东西伸延”城市发展战略，围绕现代城市、历史文化、山水生态三条城市发展主轴线，以前所未有的力度推进城市基础设施和公共设施建设，城乡面貌发生翻天覆地的变化，再度荣获“中国人居环境范例奖”。

　　城乡规划科学有序。编制完成城市总体规划、市区综合交通规划、市域城镇体系规划等100多项城乡建设规划。结合城市主体功能区规划，修编新一轮土地利用总体规划。全面完成县区、乡镇总体规划修编，全市50户以上村庄建设规划完成71.4%，形成了多层级、广覆盖的城乡规划体系。

　　城市设施全面升级。惠州大道、惠南大道、金山大道、合生大桥、惠博快速通道、金龙大道等一大批城市主干道建成通车，江北核心区、南部新城和东部新城建设快速推进，市区建成面积扩大近一倍，形成现代城市组团式发展的格局。科技馆、博物馆、文化艺术中心、会展中心、省运会体育场馆等一大批标志性建筑和挂榜阁、合江楼、丰渚园、东坡园、叶挺纪念园、邓演达纪念园等一大批文化新景点建成，丰湖书院改建、红花湖景区建设等西湖美化工程和水东街改造工程加速推进，城市功能和整体形象大幅提升。

　　城市生态更加优美。“833”生态工程深入实施，全市森林覆盖率提高到59.6%，城市人均公共绿地15.2平方米，顺利通过国家园林城市复查，荣获省林业生态市称号。红花湖环湖绿道、大亚湾绿道全面竣工，省立绿道惠州段全线贯通，建成绿道网305公里。环保力度进一步加大，五年累计否决不符合环保要求项目1003宗，淘汰落后水泥产能488万吨、钢铁产能110万吨，关停小火电机组25万千瓦，建成污水处理厂24座，市区生活垃圾无害化处理率达到100%，环保责任考核连续三年名列全省前茅。

　　四、《规划纲要》深入实施，融入珠三角实现新跨越

　　以对接珠三角 “五个一体化”为重点，在深入实施《规划纲要》中加快融入珠三角一体化。积极落实深莞惠各项合作协议和联席会议确定的各项工作，深莞惠经济圈建设加快推进。

　　交通对接取得重大成果。惠深沿海高速、潮莞高速惠州段建成通车，五年新增高速公路97.4公里，完成改扩建50.8公里，高速公路通车里程跃居全省第二；改造国道省道189.3公里、县道232.8公里，边界“断头路”基本打通，新增公路通车里程3642公里。惠大铁路二期建成通车，厦深铁路惠州段、莞惠城际轨道建设顺利推进。惠州港首条国际集装箱航线开通，沿海港口总吞吐能力达9200万吨，是“十五”期末的2.5倍。

　　产业合作取得明显成效。以实施“双转移”战略为契机，莞惠联手共建惠东、龙门两大产业转移园，签约入园项目128个，建成项目71个，总投资192.6亿元。TCL集团与深圳市政府共建华星光电8.5代液晶面板生产线。深惠两市合建的服装生产加工及物流仓储园区、谟岭新型产业园等项目顺利推进，“坪新清”产业合作示范区规划建设取得阶段性进展。旅游业联动发展，实现旅行社导游异地带团、旅游投诉异地受理。金融合作发展迅速，招商、民生、光大、东莞等股份制商业银行进驻惠州。

　　公共服务合作务实推进。大力推进深莞惠“十大对接”，共同编制《深莞惠区域协调发展总体规划》等5个专项规划。联手实施跨界河流域综合整治，完成淡澳河惠阳段综合整治工程。推行车辆通行费年票制，境内普通公路收费站全部取消。深莞惠三市的警务协作、工商管理、人才交流、住房公积金互贷、养老保险关系接转等社会管理和公共服务合作更加紧密。

　　五、改革开放全面深化，发展活力增强实现新跨越

　　重点领域改革取得新突破。完成政府机构“大部制”改革，市政府工作部门由35个调整为32个。启动并稳步推进富县强镇事权改革和事业单位人事制度改革。五年累计缩减行政审批事项350项，在全省率先实现行政许可“零收费”。投融资改革扎实推进，政府非经营性投资项目“代建制”、国库集中支付、政府采购等制度改革全面完善。国有企业改革稳妥推进，五年完成国有企业改制46户，171家劣势企业退出市场，完成德赛集团收购惠州西门子外方股份。集体林权制度主体改革基本完成。

　　外经贸转型升级步伐加快。综合运用鼓励出口政策措施，全力支持企业扩大出口，五年累计完成出口822.5亿美元，一般贸易出口、高新技术产品出口比重分别由2005年的6.2%、41.4%提升到14.4%和50.9%。200多家来料加工企业成功转型。连续四年举办高规格的招商引资推介会，累计签约项目1300多宗，签约金额超过5500亿元。五年累计新增外商投资企业1867户，累计实际吸收外商直接投资64.7亿美元，利用外资质量进一步提升。大胆探索“走出去”，在境外投资设立企业15家，投资额1.2亿美元，发展空间进一步拓展。

　　惠港台合作迈上新台阶。积极参与CEPA和泛珠三角区域经济合作，成为广东省对港澳服务业开放先行先试重点城市。香港和记黄埔参与惠州港建设，汇丰银行、东亚银行进驻惠州，粤港惠选定仲恺高新区为试点，联合推进科技创新合作，50多家企业获得香港研发基金支持。组团参加“台湾·广东周”活动，签订采购合同金额6.3亿美元，惠台经贸、农业、文化、科技交流进一步加强。

　　民营经济迅速发展壮大。五年累计新增私营企业2.4万家、个体工商户10.3万户，新认定省级民营科技企业66家。侨兴、纳伟仕、源东、富绅4家民企入选省百强民营企业，亿纬锂能、雷士照明等一批科技型民企迅速崛起。民营经济实现增加值614.2亿元，增长16%，创税135.1亿元，增长59.6%。民营企业对经济社会发展的贡献率明显提高。

　　六、“三农”工作成效显著，统筹城乡发展实现新跨越

　　按照“十个一”路线图推进城乡统筹发展，财政投入向农村倾斜，基础设施向农村延伸，公共服务向农村拓展，全市财政五年投入“三农”资金累计达到150亿元，有力推动了农村发展、农业增效、农民增收。

　　现代农业产业化能力明显提升。现代农业“5511”工程扎实推进，农业结构进一步优化，八大特色农产品种植面积达到195万亩，实现农业增加值103.6亿元，增长5.5%。五年新建现代农业示范基地68个，建成国家级粮食高产创建示范片10个，新增省级以上农业龙头企业13家、广东省名牌农产品11个，农业科技贡献率达54.5%。成功举办两届农博会，合同销售金额115.4亿元，特色农业品牌效应进一步突显。

　　农村宜居宜业环境逐步形成。行政村公路硬底化改造任务全面完成，农村客运服务均等化实现“三个100%”。建成农村饮水安全工程129宗，惠及农村人口36.7万人。农村“三清三化”、“五改五建”系列工程有序推进，新建或改建村卫生站1052间、镇卫生院73间，行政村全部实现“一村一卫生站一医生”。改造农村危房10655户，农村“一保五难”问题基本解决。完成182宗病险水库除险加固工程、90公里主要江海堤围加固工程，惠州大堤达标加固工程达到百年一遇的防洪标准，城乡水利基础设施全面完善，防灾减灾能力全面提升。

　　扶贫“双到”工作取得明显成效。扶贫开发模式不断创新，实施“双项目”扶贫方法，集中资金建设扶贫项目，帮扶单位开发致富项目，形成了贫困村脱贫致富的“双保险”机制。落实帮扶资金9500多万元，帮扶贫困村脱贫项目274个、贫困户脱贫项目5000多个，创造了扶贫开发“横河速度”的成功经验，全市74个省级贫困村率先全部脱贫。

　　七、惠民力度持续加大，民生质量改善实现新跨越

　　五年累计投入超过300亿元，完成民心工程项目101个，办成了一系列关系群众切身利益的好事、实事，获评“中国十佳最具幸福感城市”和“人民满意城市”，广东省省情调查研究中心发布的调查报告显示，惠州居民幸福感居全省第一。

　　人民生活水平大幅提升。城镇居民可支配收入23565元，增长10.7%，年均增长9.6%；农民人均纯收入8872元，增长17%，年均增长13.5%，实现连续4年两位数增长，连续5年超过城镇居民人均可支配收入增幅，城乡居民收入差距由3.17︰1缩小到2.66︰1。全市最低工资标准由410元提高到810元。五年新增就业岗位31.6万个，转移农村富余劳动力21万人，城镇登记失业率控制在2.7%以内。劳动者合法权益得到切实维护，和谐劳动关系不断发展。

　　社会保障实现城乡全覆盖。在全省率先实现城镇职工医保、居民医保和农村合作医疗“三网合一”，全面实现全民医保。养老保险实现市级统筹，农村养老保险快速推进。城乡低保、五保供养实现应保尽保，城乡低保标准自然增长机制、低保人员生活补贴与物价指数联动机制更加健全。养老、工伤、失业和生育等各项保险参保范围逐年扩大、待遇水平逐年提高。社会救助体系进一步完善，社会福利和慈善事业进一步发展。保障性住房建设加快推进，五年建成保障性住房5153套。

　　和谐平安惠州建设成效突出。以迎奥运、迎亚运、迎省运为契机，持续开展声势浩大的“东江亮剑”严打整治专项行动，依法严厉打击“两抢一盗”等各类刑事犯罪活动，建成市区社会治安视频监控系统，荣获“全国社会治安综合治理优秀市”称号。严厉打击走私贩私、制假售假和非法传销活动，产品质量和食品药品安全专项整治取得明显成效。安全生产监管进一步强化，安全生产形势保持总体稳定。城市管理标准化、规范化水平提高，“六好”平安和谐社区创建活动广泛开展，安全小区覆盖面进一步扩大。建成综治信访维稳中心（站）1774个，社会矛盾得到及时有效化解。信访工作机制不断创新，信访工作走在全省前列。

　　援建三江乡任务出色完成。投入援建资金2.1亿元，提前完成社会事业、产业恢复、农房重建、基础设施等46个援建项目，创造了三年任务一年完成的“惠州速度”。三江乡成为汶川地震灾区第一个恢复重建项目整体竣工的乡镇，被国家和省作为典型示范，展示高效高质的灾后重建成果。

　　八、两大盛事圆满完成，社会事业发展实现新跨越

　　聚全民之智，举全市之力，以全国地级市总分第一名的成绩成功摘取 “全国文明城市”金牌，圆满成功举办第十三届省运会、第六届省残运会，推动城市文明的升级提前十年、城市发展的进程提前十年。本届政府向全市人民承诺的“三件大事”已完成两件，五年经济总量再造一个惠州的目标胜券在握。

　　文明形象显著提升。以成功创建全国文明城市为新起点，文明城市创建常态化工作机制建立完善，文明成果向县城、社区、农村持续延伸，群众性精神文明活动广泛开展，城市文明程度进一步提升。“老客家，新客家，来到惠州就是一家；本地人，外地人，在惠州工作就是惠州人”成为惠州的城市品格，“崇文厚德、包容四海、敬业乐群”的新时代惠州精神深入人心。在支援汶川、玉树抗震救灾，迎接奥运、亚运火炬传递等一系重大活动中，充分体现了惠州人民的高尚情怀和文明素质。

　　省运盛会精彩圆满。以精彩绝伦的开闭幕式、科学周密的竞赛组织、顺畅高效的场馆运行、热情周到的赛事服务、万无一失的安全保障，成功举办第十三届省运会和第六届省残运会，实现“四个一流”办赛目标，赢得全省人民的高度好评，被誉为“广东空前、全国一流”的一届体育盛会，全面展示惠州文明和谐的新形象、科学发展的新成就和宜居宜业的新魅力。

　　成功创建省教育强市。城乡教育优先均衡发展，在全省率先实现城乡教师待遇“两持平”，全面解决代课教师问题。城乡全面实现九年免费义务教育。华罗庚中学等一批新校建成招生，惠州学院新校区建设完成，技师学院、中职新城等新校建设加快推进。五年新增中小学学位6.6万个、中职学位3.8万个，高中阶段教育毛入学率达到90.6%，提前两年高分通过“普高”验收，提前一年成功创建省教育强市，被确定为全国推进义务教育均衡发展试点城市。

　　文化惠州建设扎实推进。五年累计建成市级文化场馆13个、县级文化场馆70个、镇级文化站68个、“农家书屋”730家，覆盖城乡的公共文化服务设施网络基本形成。群众性文化活动蓬勃开展，“快乐时光”等群众文化活动成为独具特色的文化品牌。文化体制改革深入开展，成立市国有文化资产运营公司，组建报业传媒集团、广电传媒集团。惠东渔歌被列入国家第二批非物质文化遗产名录，龙门县以农民画入选中国民间文化艺术之乡，惠阳秋长被列入第四批国家级历史文化名镇。创作《悠悠东江情》等一批文艺精品，在全省“五个一工程”评选中获奖数量连续四届名列全省地级市首位。

　　社会各项事业取得新进步。医药卫生体制改革不断深化，启动公立医院改革，全面实施基本药物制度。新建市第一人民医院、第一妇幼保健院，市中心人民医院改造和中医医院新院建设进展顺利，基层医疗卫生服务覆盖率达到100%。人口与计划生育工作连续15年获评全省先进，荣获全国人口计生综合改革示范市。顺利完成经济和人口普查。第三次获得“全国双拥模范城”称号。物价、气象、地震、移民、口岸、妇女儿童、外事侨务、港澳台事务、友好城市等工作取得新成效。民族宗教、国防教育、人防、消防、应急、民兵、档案、方志、仲裁、老龄、残疾人、红十字、老区建设等工作取得新进展。

　　民主法制和政府自身建设进一步加强。自觉接受人大的法律监督和政协的民主监督，定期向市人大报告、向市政协通报工作。五年共办理人大代表议案8件、建议635件，政协提案841件，办复率100%。全面完成“五五”普法，法治城市创建活动深入开展。依法行政、廉政建设、政务公开全面推进，审计、监督工作力度加大。专家咨询论证、社会公示和社会听证等制度进一步完善，万众评公务、网络问政、“行风热线”、“惠民在线”形成常态化机制。学习型、服务型政府全面建立，市行政服务中心高效运作，机关服务质量、服务效能显著提升。

　　各位代表，回顾“十一五”发展历程，成绩来之不易，奋斗充满艰辛，经验弥足珍贵。我们深刻体会到：一是必须坚定不移坚持以科学发展观统领全局，推动经济与社会协调发展、城市与农村统筹发展、人与自然和谐发展，提升发展的质量和水平，促进发展方式加快转变。二是必须坚定不移坚持创造性地开展工作，尤其是面对国际金融危机等重大挑战时，创新工作思路和举措，以敢为人先的精神攻坚克难，在奋力拼搏中开创发展的新局面。三是必须坚定不移坚持大气魄、大动作、大手笔的发展格局，围绕《规划纲要》实施，以世界眼光和战略思维谋划惠州的未来发展，加快融入珠三角一体化，进一步拓宽发展空间，提升惠州在珠三角的城市地位。四是必须坚定不移坚持重大项目带动战略，把谋划建设重大项目作为扩内需、调结构、促转变、惠民生的重要支撑，进一步增强经济实力和发展后劲，为建设科学发展的 “惠民之州”奠定坚实的物质基础。五是必须坚定不移坚持改革开放，全面推进体制机制改革创新，坚决消除阻碍发展的体制机制束缚，增强发展的动力和活力，同时努力扩大对外开放，强力推进招商选资，在更大范围、更高层面集聚发展资源，推动经济社会快速发展。六是必须坚定不移坚持以民为本，把改善民生作为发展的根本目的，用发展的成果促进民生的改善、提升民生的质量，全面推进和谐社会建设。这些认识和体会，是惠州科学发展、加快发展的宝贵经验，我们将继续传承和发扬。

　　五年来，全市人民用勤劳和智慧谱写了惠州科学发展的辉煌篇章。今天的惠州，已经成为世人瞩目、充满活力的生态丽城、宜居名城、工业新城、文明城市。在此，我代表惠州市人民政府，向为惠州发展付出辛勤劳动、作出积极贡献的全市干部群众、外来务工人员、海内外投资者，表示崇高的敬意！向给予政府工作大力支持的人大代表、政协委员、各民主党派、工商联和社会各界人士，向关心支持惠州建设的驻惠部队、武警官兵、中央和省驻惠单位、港澳台同胞、海外侨胞、国际友人，表示衷心的感谢！

　　在肯定成绩的同时，我们也清醒地看到前进道路上还存在不少困难和问题：现代服务业发展相对滞后，战略性新兴产业发展仍处于起步阶段，加快产业优化升级的要求更加迫切；自主创新能力不强，节能减排任务艰巨，加快转变经济发展方式的要求更加迫切；区域、城乡发展还不平衡，山区、贫困地区的发展较为缓慢，全面推进区域协调发展、城乡统筹发展的要求更加迫切。上述问题必须引起我们高度重视，并在今后的工作中切实加以解决。

“十二五”时期的形势、目标和任务

　　“十二五”时期，是我市实现科学发展新跨越的战略机遇期，也是我市瞄准先进科技、加快转型升级的全面攻坚期。“十二五”时期，国内外宏观环境将继续发生深刻变化。从国际环境看，经济全球化深入发展，全球经济格局深度调整，在逐步走向复苏的同时存在诸多不确定、不稳定因素，特别是各种形式的保护主义抬头，对外向度较高的惠州经济影响将更为深刻。从全国来看，我国经济仍处于快速成长期，工业化、城镇化步伐继续加快，消费需求不断扩大，经济结构加快转型，体制活力显著增强，社会大局保持稳定，为我市经济发展创造了优越的条件。从全省来看，省委、省政府实施了一系列扩大内需战略、自主创新战略、区域协调发展战略、绿色发展战略，着力推进高新技术产业、先进制造业、现代服务业和战略性新兴产业的发展，为我市加快经济转型升级注入了强大动力。从我市来看，经过“十一五”时期的快速发展，经济综合实力明显增强，现代产业体系基本形成，一大批重大项目动工建设和建成投产，为加快发展奠定了坚实基础，积蓄了强劲势能和后劲。尤其是 《规划纲要》的深入实施，为我市经济社会发展提供了更加广阔空间。综合判断，“十二五”期间，我市既面临着诸多的考验和挑战，也面临着难得的发展机遇，更具备了加快发展的雄厚实力。我们必须进一步增强推进科学发展的自觉性和坚定性，进一步增强转型升级的机遇意识和忧患意识，进一步增强建设惠民之州的责任感和紧迫感，以更加高昂的斗志、更加务实的作风、更加有力的举措、更加扎实的工作，全面开创我市科学发展的新局面，创造惠州“十二五”发展的新辉煌。

　　“十二五”时期，我市经济社会发展的指导思想是：坚持以邓小平理论、“三个代表”重要思想为指导，深入贯彻落实科学发展观，全面贯彻党的十七届五中全会、省委十届八次全会和市第九次党代会第五次会议精神，积极实施《规划纲要》，以科学发展为核心主题，以结构调整为主攻方向，以科技创新为重要支撑，以改革开放为强大动力，以改善民生为根本目的，加快转型升级，升华惠民之州，为率先建成幸福的惠民之州奠定雄厚的基础。

　　今后五年，我市经济社会发展的主要目标是：生产总值年均增长10%以上，人均生产总值年均增长8%以上。高新技术产业增加值占规模以上工业增加值的比重达到45%以上，服务业和战略性新兴产业增加值分别占生产总值的36%、8.5%，建成现代产业100强。五年累计新增就业岗位32.5万个，转移农村富余劳动力6.5万人，城镇登记失业率控制在3%以内。城乡居民人均收入年均增长10%以上。九年义务教育巩固率达到96%，高中阶段教育毛入学率保持在90%以上。夺取“全国文明城市标兵”金牌。

　　做好“十二五”工作，实现“十二五”目标，重点是推动“六个提升”：

　　一是提升发展主题，创新幸福成果。把“加快转型升级，升华惠民之州，建设幸福惠州”作为落实科学发展观的重要目标和战略举措，作为贯穿“十二五”时期工作的重中之重和“头号工程”，以更新的理念、更高的标准、更优的质量，推动经济社会又好又快发展，努力为人民群众创造更多的幸福成果。

　　二是提升“八大基地”，夯实惠民基础。以构建具有国际竞争力的高端化、集群化、总部型、创新型的现代产业体系为目标，全力提升“八大基地”建设水平，推动现代产业规模进一步壮大、实力进一步增强，以产业的升级促进经济的升级，以经济的升级保障民生的升级。

　　三是提升文化软实力，建设文化惠州。加快完善城乡公共文化服务体系，深入挖掘岭南文化和客家文化资源，打造更多具有惠州特色、反映时代风貌的文化品牌，大力推动文化产业集约化、规模化、品牌化发展，努力把惠州建设成为文化惠民先行区、文化产业新兴区和粤港澳地区重要的文化城市。

　　四是提升民生质量，增加幸福指数。坚持新增财力70%以上用于民生事业，大力发展劳动就业、社会保障、医疗保险、住房保障、教育、文化等各项民生事业和社会事业，推动民生事业从低标准的普惠型向高标准的福利型升级发展，实现民生改善与经济发展同步共进，幸福指数与发展指数同步提升。

　　五是提升社会管理，增加和谐指数。进一步健全文明城市建设长效机制，丰富和拓展文明城市创建内涵和外延。完善信访、调解、综治三位一体的矛盾纠纷排查调处机制，及时有效化解社会矛盾。加大社会管理力度，全面建设平安惠州，让惠州发展的环境更加优越、文明的社会更加和谐。

　　六是提升生态环保，增创环境优势。始终坚持生态优先原则，大力推进“833”生态工程和区域绿道网建设，巩固和提升生态环境建设成果。大力发展循环经济和低碳经济，建设资源节约型、环境友好型社会。在经济快速发展中长久保持惠州山清水秀的生态竞争力，让人民群众充分享受到在环境优美城市居住的幸福。

2011年工作安排

　　2011年，既是实施“十二五”规划的开局之年，又是本届政府任期的最后一年，对于圆满完成本届政府各项工作任务，实现“十二五”规划高起点开局至关重要。政府工作的基本思路是：全面贯彻党中央、国务院和省委、省政府的决策部署，按照市第九次党代会第五次会议的总体安排，突出抓好产业升级和自主创新，推动发展方式率先转变；突出抓好扩大内需和招商引资，确保经济持续稳定增长；突出抓好《规划纲要》实施和“双转移”，加快融入珠三角一体化；突出抓好城市轴线和区域绿道建设，打造现代化“双宜”城市；突出抓好现代农业和扶贫“双到”，促进城乡统筹发展上新台阶；突出抓好惠民工程和社会事业，推进民生质量进一步改善，全力确保“十二五”规划开局良好，努力争当加快转型升级的排头兵，迈出建设幸福惠州更加坚实的步伐。

　　综合考虑各方面的因素，2011年全市经济社会发展的主要预期目标是：生产总值增长11%；人均生产总值增长9%；节能减排完成省下达任务；固定资产投资增长8%；外贸出口增长7%；地方财政一般预算收入增长13%；社会消费品零售总额增长15%；居民消费价格指数控制在104%以内；服务业增加值占GDP的35%；全社会研究与开发经费支出占GDP比重完成省下达任务；城镇登记失业率控制在2.7%以内；户籍人口自然增长率控制在8.6‰以内；城市居民人均可支配收入增长10%；农民人均纯收入增长10.5%。

　　实现上述目标，要努力在调结构中促转型，在促转型中稳增长，在稳增长中惠民生，以更大力度推进八项重点工作：

　　一、以更大力度调整产业结构，加快产业优化升级

　　进一步壮大石化产业集群。大力推进中海油二期1000万吨炼油、100万吨乙烯项目建设，开工建设中海油ABS项目、深圳彩虹精细化工项目等一批石化重点项目。积极引进“隔墙供应”项目和石化中下游项目、精细化工项目，延伸石化产业链，推进大亚湾精细化工园区建设，加快形成规模化、集聚化发展的石化产业集群。认真抓好大亚湾石化区填海扩区工作，拓展产业发展空间。

　　加快发展高端电子信息产业。加快TCL液晶产业园三期、LG伊诺特LED封装等项目建设，推动TCL8.5代液晶面板扩大产能，打造全球最大的液晶电视生产基地。全力推进清华同方现代信息服务基地、龙旗通信手机研发等项目建设，加快发展新型显示、新一代通信、数字装备制造等高端电子产业。

　　推动清洁能源产业快速发展。积极推进惠州国家石油储备基地、国华热电联供二期等项目建设，启动LNG电厂二期和丰达电厂扩建工程，做好中广核惠州核电项目前期工作，确保抽水蓄能电站全面投产。完成省天然气管道工程惠州段、西气东输二线惠州段、市区燃气管道建设。

　　大力发展战略性新兴产业。抓紧编制战略性新兴产业发展规划，制订出台培育战略性新兴产业的政策措施。积极推进省市共建战略性新兴产业基地建设，依托科锐光电LED芯片项目，做大做强LED产业，打造LED产业基地和LED绿色照明示范城市。大力扶持入选省现代产业500强的37个项目和市现代产业100强项目，促进生物医药、新材料、节能环保等战略性新兴产业加快发展。

　　着力提升旅游业发展水平。继续深入实施旅游业发展“3510”工程，大力推进环大亚湾滨海旅游度假区、环大南昆山生态旅游度假区建设，抓好西湖、罗浮山景区创5A、龙门天然温泉景区创4A工作，加快发展高端旅游业，力争旅游接待总人数、旅游总收入分别达到2750万人次、160亿元。

　　积极扩大现代服务业规模。推进惠州港国际集装箱码头、石化区公用货运码头、荃湾港区主航道扩建等项目建设，加快发展港口物流业。大力培育3G信息、会展经济、文化创意、工业设计、总部经济等产业。加快推进入选首批省级现代服务业集聚区的3个项目和仲恺高新区总部经济大楼建设。积极发展现代金融业，力争引进3家银行，引导5家企业上市。

　　二、以更大力度扩大内外需求，增强经济发展动力

　　促进投资增长。全力推进一批关系长远、带动全局的基础设施、高新产业、新兴产业、社会民生等重大项目建设，不断增强经济发展的后劲。全年安排重点项目建设144项，总投资3169亿元，年度计划投资337亿元。其中新开工项目37项，续建项目85项，投产项目22项。做好重点项目储备，促进重点项目建设有序接替、梯次滚动发展。

　　扩大消费需求。继续落实家电下乡、以旧换新等政策措施，积极推进“万村千乡”市场工程。不断拓展文化娱乐、体育健身等消费热点。总结提升 “惠货全国行”经验，举办南宁、郑州、重庆三场大型展销会。鼓励支持企业在国内大中城市开设品牌店、专卖店、商贸城，建设“惠州产品网上展销会”等公共服务平台，努力扩大内销市场。

　　强化招商选资。举办大型招商引资推介会，大力引进世界500强企业、跨国集团、大型央企省企、国内100强企业和成长型、技术型新兴产业项目。进一步完善园区基础设施和配套设施，打造承接产业强势载体。积极推进产业招商和重大项目招商，做到引进项目与引进技术、管理、人才同步推进，吸引各类高端人才、创新人才。做好对现有企业的服务，促进企业增资扩产、延伸发展。

　　优化出口结构。坚持重点企业联系制度，大力帮扶出口大户扩大外贸出口。组织企业参加广交会、高交会、中国-东盟博览会等系列经贸活动，巩固传统市场，拓展新兴市场。大力支持自主知识产权、自主品牌、自主营销商品出口，扩大机电和高新技术产品出口。继续推进来料加工企业转型升级。

　　壮大民营经济。认真落实扶持民营经济发展各项政策措施，鼓励民营资本参与基础设施建设、新兴产业发展。切实帮助民营企业解决生产经营中遇到的困难，完善民营企业融资服务体系。深入实施“百十亿”民营企业培育工程和民营企业家培训工程，培育一批博士民营企业家。支持民营企业开展技术创新、自营出口，推动民营经济转型升级。

　　三、以更大力度推进科技创新，提升自主创新能力

　　完善科技创新体制机制。积极实施“双提升”战略，全面落实科技创新政策，进一步完善以企业为主体、市场为导向、产学研相结合的技术创新体系。认真执行科技投入政策规定，切实加大科技投入。通过基金支持、创业投资、贷款贴息、税收优惠等方式，扶持企业开展技术创新活动。力争科技对全市工业经济增长贡献率达到57%。

　　推进科技创新平台建设。大力推进“省部产学研结合示范市”建设，抓好仲恺电子信息研究院、大亚湾石油化工研究院等共性技术研发平台建设，加快创建专业镇技术创新平台。加强与高校和科研院所的科技对接，鼓励企业与国内外科研机构开展科技合作，大力实施惠港联合推进高新区发展合作项目，支持TCL集团与中山大学共同组建工程中心，力争组建产业技术创新联盟1个，新增市级以上工程中心（农创中心）12家。

　　加快发展高新技术产业。推进东江高新技术产业园、大亚湾科技创新园建设。大力引进高新科技项目，推动高新技术产业快速发展，打造高端产业区域。支持TCL、德赛、华阳、侨兴等企业进入广东“自主创新100强”。力争新认定高新技术企业15家、国家级创新型试点企业1家，新培育民营科技企业20家，高新技术产品产值增长10%以上。

　　加强科技孵化器建设。重点加快省级以上创业中心、留学人员创业园、软件科技园等孵化器建设，完善孵化功能，加快形成孵化集群，为创业企业打造孵化平台。深入开展质量强市活动，推广质量管理先进技术。积极实施名牌战略，加强专利申报工作，力争专利申请量、授权量分别增长20%。

　　四、以更大力度落实《规划纲要》，加快融入珠三角一体化

　　提升“五个一体化”档次和水平。加快编制深莞惠区域协调发展规划、交通一体化规划，着力抓好仁深、从莞、深惠等高速公路惠州段建设，确保广河高速惠州段通车。积极推进厦深铁路、莞惠城际轨道建设，认真做好轨道交通深惠线、广州至汕尾铁路惠州段前期工作，着力构建与珠三角无缝对接的和谐交通大体系，加速融入“珠三角一小时生活圈”。大力推进公共服务一体化合作，力争在公交一卡通、医保一卡通等方面取得新进展。

　　积极参与深莞惠经济圈建设。全面加强产业合作，大力推进惠东、龙门两个省级产业转移园建设，启动“大亚湾-坪山”新能源产业合作示范区和“坪新清”产业合作示范区建设。继续深化水利、环保合作，协调推进界河及跨界河综合治理。强化环境污染联防联治，促进公共设施共建共享。加快推进三市医保服务系统互联互通、劳动用工联动招聘、异地养老服务，加快实现三地通信资费统一、图书馆资源共享等，进一步深化三市公共事务合作。

　　大力拓展惠港合作领域。积极推进国际集装箱码头、城轨交通一号线等惠港合作项目的建设和筹备，力争恢复惠州港与香港的海上直航。推动惠港科技合作深入开展，力争香港生产力促进局在惠设立机构。全面加强惠港服务业合作，大力引进香港金融服务、港口物流、文化创意、工业设计等现代服务业。积极引进港资兴办现代农业龙头企业，构建惠港两地农产品长期供给关系。进一步加强教育、医疗、会计、律师、社会管理和民政事业等领域的合作，共建优质生活圈。

　　五、以更大力度统筹城乡发展，促进城乡共同繁荣

　　推动现代农业转型升级。继续实施现代农业“5511”工程，大力发展特色效益农业，打造一批连片面积5万亩以上的特色产业带，力争全市八大特色农产品种植面积达200万亩以上，全面实现现代农业“八大目标”。培育一批农业品牌产品，新增省市名优产品5个。积极发展农业龙头企业，新增市级以上农业龙头企业11家。提高现代农业技术装备水平，农业科技贡献率达到56%，农业机械化水平达到63%。推动现代农业与旅游业相结合，发展农家乐、观光农业等旅游项目。

　　加强农村基础设施建设。坚持财政支出重点向农业农村倾斜，进一步完善农村水利、道路、通信、公交等基础设施。启动建设惠州大堤 （南堤）堤路贯通工程，推进马安围平马围合围安全加固工程、东江水利枢纽库区防洪排涝整治工程，完成38宗病险水库和4宗中型水库除险加固工程。完成规划内农村饮水安全工程建设任务。全市90%以上行政村实现“七通八有”。

　　进一步深化农村综合改革。全面推进富县强镇事权改革，完善县、镇经济社会管理体制。坚持最严格的耕地保护制度，深化征地制度改革。推进农村产权改革，促进土地承包经营权有序流转，全面完成集体林权制度改革。切实抓好镇、村“两委”换届选举工作，加强基层干部队伍建设，增强农村组织保障能力。

　　高标准完成扶贫“双到”任务。落实扶贫责任，加大扶持力度，动员社会力量，共同推进扶贫“双到”工作。组织农业龙头企业与贫困户结对帮扶，提高贫困户种养能力，带动贫困户增收，实现稳定脱贫。加大农村劳动力转移就业力度，推进扶贫开发“户户有就业”工程。大力实施扶贫项目，确保扶贫“双到”任务高标准提前完成。

　　六、以更大力度推进城市建设，打造珠三角城市群明珠

　　科学规划空间布局。以打造广东最优美的宜居城市为目标，完善城市功能区规划，优化市域城镇和城市内部空间布局，科学规划全市人口、产业、生态、交通和重大基础设施、公共设施布局。继续实施“南进北拓、东西伸延”城市发展战略，大力推进江北核心区、南部新城、东部新城、中职新城、惠博新城等城市组团建设，加快形成以现代城市、历史文化、山水生态三条城市主轴线为架构的城市发展新格局。

　　全面提升城市功能。实施一批市区交通顺畅工程，启动云山东江大桥建设，加快推进四环路建设、三环路改造、仲恺大道改扩建等工程，完成惠州大道东段改造、西枝江大桥改造，全面推进城市主干道延伸和升级。大力推进市民乐园地下商城建设，整合华贸中心、会展中心、文化艺术中心，加快形成珠三角核心商务区。以更有效的手段大力推进“三旧”改造，加快水东街、丰湖书院等改造工程和“岭南新民居”示范点建设，进一步提升人居环境质量。加快推进绿道建设，完善省立绿道的路标、驿站等配套设施和绿化植被，建设中职新城至三环路等城市和社区绿道，启动县区级绿道网规划建设工作。

　　积极创建全国文明城市标兵。推进文明城市常态化建设，加快建立城市管理数字化信息系统，提高城市管理能力和水平。扩大文明创建覆盖面，推动文明创建向县镇、农村、社区延伸，评选一批文明示范村镇和文明社区标兵。组织开展“百字系列”现代公民道德教育活动，大力弘扬新时代惠州精神，营造积极向上、团结和谐的社会氛围。认真做好各项“迎检”准备，确保高分通过全国文明城市复检，实现蝉联全国文明城市目标。

　　优化城乡生态环境。继续推进“833”生态工程，力争森林覆盖率达到59.8%。启动国家生态市创建工作和碳汇林业项目建设，实施西湖生态修复与水质改善工程，修复潼湖万亩湿地、范和港湿地，加快惠州海洋生态园建设，打造红树林建设示范工程。推进城乡绿化一体化工程建设，新增城市公共绿地80万平方米，市区人均公共绿地面积达到15.5平方米。推进龙门县创建国家级生态县，全市创建4个生态乡镇、22个生态示范村、7个宜居城镇、14个宜居村庄、50个宜居社区。

　　进一步加强节能减排。强化节能监察，狠抓工业、交通、建筑、商贸酒店、公共机构等重点领域节能。加快关停能耗大、污染重的企业，淘汰落后产能，确保完成省下达的节能任务。大力推进中心镇、淡水河流域、西枝江流域和潼湖流域污水处理厂建设，新建污水处理厂9座，全市城镇生活污水处理率达到75%以上，确保顺利通过国家环保模范城市复查。大力推进节约集约用地，盘活存量用地，提高土地利用效率。大力发展低碳经济，制订推广低碳生活消费计划，努力争当低碳发展的先行区。

　　七、以更大力度建设文化惠州，推动社会和谐发展

　　提升教育发展水平。全面巩固教育“创强”成果，推动教育事业优先、优质、均衡、快速发展。高度重视发展学前教育，加快镇、村幼儿园建设。整合城乡教育资源，完善城乡中小学校标准化建设，深入开展义务教育均衡发展试点工作。加快县（区）标准化特殊学校建设。实施现代学校制度，提升素质教育水平。做大做强职业教育，加快推进中职新城建设，确保技师学院、卫生高级职业技术学校、商贸旅游高级职业技术学校一期建成。支持惠州学院、经济职业技术学院发展。设立扶持民办教育专项基金，鼓励支持社会力量办学。

　　大力发展文化事业产业。新建县（区）文化馆、图书馆、博物馆各1个，改造建设镇 （街道）综合文化站73个、村（社区）文化室115个，加快形成覆盖城乡的公共文化服务网络。深入开展文化下乡、送书下乡等文化惠民活动，组织公益性文艺演出进农村（社区）、进企业。挖掘优秀岭南文化、客家文化资源和民族民间艺术，创作一批文化精品，打造一批特色文化品牌。加强文化遗产保护和修复，推进国家历史文化名城创建工作。积极发展新闻传媒、文化创意、数码出版、会展博览、演艺娱乐等文化产业，加快推进粤港澳影视拍摄基地、惠州报业全媒体产业基地等文化产业项目建设，大力培育迪可可国际儿童城、天后宫岭南民俗文化街等文化旅游项目，壮大文化产业规模，推动文化产业升级。

　　加快发展卫生等各项事业。深化医药卫生体制改革，推进公立医院改革试点，全年安排医改资金1.28亿元。继续推进“健康惠州”工程，提高城乡公共卫生服务水平。加快推进市中心人民医院改扩建和市中医医院新院等工程建设，全市80%以上社区卫生服务中心、70%以上镇卫生院、60%以上村卫生站达到标准化。以成功举办第十三届省运会为新起点，广泛开展全民健身活动，推动体育事业加快发展。加强人才培养、引进和管理，壮大人才队伍。深化人口计生综合改革，确保完成省下达的计生任务。加强民兵预备役建设，深入开展“双拥”和优抚安置工作，巩固军政、军民团结，确保夺取“全国双拥模范城”四连冠。

　　创新和加强社会管理。深入持久开展“东江亮剑”专项行动，严厉打击违法犯罪活动，提高社会治安综合治理水平。全面实行党政领导干部安全生产 “一岗双责”，强化和落实企业安全生产主体责任，加强安全生产监管、隐患排查、综合整治，有效防范和坚决遏制各类安全事故，确保安全生产形势稳定。加强食品药品安全和产品质量管理，打击走私贩私、制假售假，进一步规范市场经济秩序。加大信访维稳和人民调解工作力度，积极回应、及时解决群众合理诉求。

　　深入推进民主法制建设。自觉接受人民代表大会及其常委会的法律监督和工作监督，依法向人大及其常委会报告工作，切实执行人大及其常委会的各项决定决议。主动加强与人民政协的联系，认真办理人大代表、政协委员的议案、建议和提案，不断提高办理质量。认真听取各民主党派、工商联、无党派人士的意见。扎实推进“六五”普法，加快建设法治惠州。

　　着力打造服务型政府。积极推进“大部制”改革，坚持依法行政，深化行政审批制度改革，建立健全县（区）、镇行政服务中心，进一步提高政府行政效能。加强政府廉政建设，坚持民主科学决策。完善政务公开，深入开展“万众评公务”活动，巩固和完善 “行风热线”、“惠民在线”等问政平台建设，进一步拓展政府与群众沟通的渠道，为人民群众提供优质高效的政务服务。

　　八、以更大力度增进民生福祉，办好十件惠民实事

　　启动新一轮惠民工程建设项目，全年安排惠民工程100项，年度投资134亿元。同时，市政府向全市人民承诺，今年将办好十件惠民实事：

　　一是加大教育惠民力度。龙门县60%的乡镇、其他县（区）90%的镇（街道）均建成1所以上中心幼儿园。全市义务教育学校100%达到规范化学校标准。惠阳区、惠东县、博罗县、龙门县各建成1所标准化特殊教育学校。财政一般预算支出中教育拨款比例比上年提高1个百分点，增加100万元教师培训经费，市财政继续安排1600万元山区教师补助经费，设立推进城乡义务教育均衡发展工作政府奖励基金，设立100万元扶持民办学校发展专项基金。扶持经济薄弱乡镇“创强”，创建省教育强镇8个以上。

　　二是加大社会保障力度。连续第七年提高企业退休人员基本养老金，企业退休人员人均养老金每月增加145元。扎实推进新型农村社会养老保险试点工作，确保惠城区、惠阳区、博罗县、大亚湾开发区、仲恺高新区年底前实现全覆盖，惠东县、龙门县20%以上乡镇开展试点。

　　三是加大医疗保障力度。医疗保险实现市级统筹，城乡居民医保限额从每年8万元提高到每年10万元。加快建立定点医疗机构实时结算制度。继续推进免费婚检工作，全市婚检率达到70%以上。

　　四是加大住房保障力度。启动江北45号小区二期、古塘坳尖峰山、火车西站等保障性住房建设项目，全市筹建保障性住房4032套，进一步解决低收入群众的住房困难。计划筹集2.3亿元，启动移民安居工程。大力推进住房公积金缴存扩面工作，提高城镇职工基本住房保障水平和住房消费能力。

　　五是加大就业工作力度。新增城镇就业岗位6.5万个、就业人员6万人，城镇登记失业率控制在2.7%以内。转移农村富余劳动力2万人。培训各类人员9万人。

　　六是加大扶贫开发力度。实施产业扶贫，力争每个贫困村发展1~2个特色产业，带动贫困户脱贫，确保2742户省级贫困户年人均纯收入达到2500元以上，实现稳定增收目标。实施转移就业扶贫，力争每一户贫困户至少转移1名劳动力，完成350名农村贫困家庭劳动力职业技能培训任务，在74个省级贫困村所在的20个乡镇举办20场扶贫招聘会。

　　七是加大福利惠民力度。把人均年收入低于2550元的困难家庭全部纳入低保范围。扩大高龄老人政府津贴发放范围，提高津贴标准，对80岁以上老人每人每月发放50元，90岁以上老人每人每月发放100元，100岁以上老人每人每月发放300元。推进综合福利院新院、社会福利服务中心、居家养老服务示范中心和市残疾人托养抚养服务基地建设。

　　八是加大惠农设施建设力度。加快推进农村饮水安全工程建设，解决规划内剩余的12.4万农村人口饮水安全问题。大力推进农村信息化建设，支持中国电信与TCL集团联手运用三网融合技术，实施“村村通光纤、户户通互联网数字电视”工程，在全省率先实现互联网全覆盖。建设通自然村公路800公里。完成贫困户危房改造2200户。

　　九是加大物价调控力度。大力推进稳定物价“三项建设”，全市安排价格调节基金2000万元，其中，500万元扶持生产基地蔬菜大棚建设，300万元支持农产品储备冷库建设，设立60家以上农产品平价门店。实施低收入群体生活补贴与价格指数、收入水平双联动机制，及时发放价格补贴及应急调控补贴。

　　十是加大公交惠民力度。优化中心城区公交线路及站点，新增公交线路15条，更新LNG公共汽车100辆，新增公共汽车200辆，新增公交运力全部投放节能环保车型。推行“岭南通·惠州通”智能卡，逐步应用于公交车、道路客运、出租车等运输行业，力争实现珠三角公交一卡通。

　　各位代表，承接“十一五”辉煌，开创“十二五”伟业，是时代赋予我们的光荣使命。让我们在省委、省政府和市委的正确领导下，深入贯彻落实科学发展观，全力以赴，奋发进取，加快转型升级，升华惠民之州，建设幸福惠州，确保“十二五”开好局、起好步，以优异的成绩迎接建党九十周年！
